# The lived neighborhood: understanding how people with dementia engage with their local environment

**DOI:** 10.1017/S1041610217000631

**Published:** 2017-05-02

**Authors:** Richard Ward, Andrew Clark, Sarah Campbell, Barbara Graham, Agneta Kullberg, Kainde Manji, Kirstein Rummery, John Keady

**Affiliations:** 1 Faculty of Social Science, University of Stirling, Stirling, United Kingdom of Great Britain and Northern Ireland; 2 School of Nursing, Midwifery, Social Work and Social Sciences, University of Salford, Salford, Greater Manchester, United Kingdom of Great Britain and Northern Ireland; 3 School of Nursing, Midwifery and Social Work, University of Manchester, Manchester, United Kingdom of Great Britain and Northern Ireland; 4 Institute for Medicine and Health, Linköping University, Linkoping, Sweden

**Keywords:** dementia, neighborhoods, environment, social networks, dementia friendly communities

## Abstract

**Background:**

In this paper, we report progress on “Neighborhoods: our people, our places” an international study about how people living with dementia interact with their neighborhoods. The ideas of social health and citizenship are drawn upon to contextualize the data and make a case for recognizing and understanding the strengths and agency of people with dementia. In particular, we address the lived experience of the environment as a route to better understanding the capabilities, capacities, and competencies of people living with dementia. In doing this, our aim is to demonstrate the contribution of social engagement and environmental support to social health.

**Methods:**

The study aims to “map” local spaces and networks across three field sites (Manchester, Central Scotland and Linkoping, Sweden). It employs a mix of qualitative and participatory approaches that include mobile and visual methods intended to create knowledge that will inform the design and piloting of a neighborhood-based intervention.

**Results:**

Our research shows that the neighborhood plays an active role in the lives of people with dementia, setting limits, and constraints but also offering significant opportunities, encompassing forms of help and support as yet rarely discussed in the field of dementia studies. The paper presents new and distinctive insights into the relationship between neighborhoods and everyday life for people with dementia that have important implications for the debate on social health and policy concerning dementia friendly communities.

**Conclusion:**

We end by reflecting on the messages for policy and practice that are beginning to emerge from this on-going study.

## Introduction

In this paper, we introduce the “Neighborhoods: our people, our places” project (N:OPOP), a five-year initiative jointly funded by the Economic and Social Research Council and the National Institute for Health Research (UK). As part of a larger program investigating dementia and neighborhoods (Keady, 2014), this qualitative longitudinal and comparative study is currently exploring the meaning and experience of neighborhood for people affected by dementia. Our aim is to better understand the lived experience of the localities in which people reside and how the onset and progression of dementia is managed in this context. The research will subsequently inform the design and piloting of neighborhood-based interventions in each of the three project fieldsites and through this we aim to translate findings from the study into practice innovations.

Our aim for this paper is to provide insights into the dynamic process whereby people living with dementia balance their capabilities and limitations as they engage with external factors, both social and environmental, as a crucial dimension of “social health.” The notion of social health has emerged as part of a broader critique of the WHO definition of health, formulated in 1948 as “a state of complete physical, mental, and social well-being, and not merely the absence of disease or infirmity” (WHO, 2006). In a context of ageing societies, where chronic illness has become more widespread, subsequent efforts to redefine health have centered upon the ability to adapt and self-manage. Huber and colleagues (2011) thereby note the significance of “social health” arguing that several dimensions of health can be identified in the social domain. These include a person's capacity to fulfill their potential and obligations, the ability to manage their life with some degree of independence and the ability to participate in social activity. Most recently, Vernooij-Dassen and Jeon (2016) have argued for the importance of recognizing the social health dimension to dementia. Here, we build on this line of argument to explore social health in a neighborhood context for people living with dementia.

We add to the debate on social health through close attention to the environmental challenges that people with dementia face in the course of their everyday lives. Our analysis and discussion underlines the importance of attending to the lived experience of place and space, which has the potential to provide vital insights into our understanding of social health and policy concerning the “dementia friendly community.” Our argument is that while social health may well prove useful as a representational category for the purposes of rethinking health policy and administration, we should also seek to make sense of it through the lens of lived experience. Hence, we focus on the ways that people with dementia engage with the actual properties and attributes of their neighborhoods in the course of day-to-day living. We offer insights into the process by which people balance their capabilities, capacities, and competencies with the environments they inhabit, accessing different forms of capital and exploiting the material and social affordances that exist at a local level to enable social health. We pay particular attention to the relationship that people enjoy with the more immediate environment that exists just beyond their front-door; a world that is familiar and well-trodden but also very much part of the public realm (Lofland, 1998). We also consider how the neighborhood overlaps with the home, whereby domestic boundaries are often experienced as permeable, highlighting the importance of also recognizing the dialogue between home and neighborhood in people's everyday lives.

## Background: neighborhoods and dementia

Neighborhoods are important to our understanding of social health not least because research has shown that following a diagnosis of dementia people experience a “shrinking world” (Duggan *et al*., 2008). The boundaries to both the social and physical environments that people routinely inhabit constrict over time and this means that the neighborhood takes on a particular significance in a context of living with dementia. According to a social health lens the neighborhood is important because it offers the most immediate opportunities for a person with dementia to participate in social activity and to fulfill their potential and obligations and is thereby a means to avoid the retreat into domestic confinement, which has been shown to compound social isolation following a diagnosis of dementia (Alzheimers Society, 2013).

Yet, until now attention to the lived experience of outdoor environments remains limited within dementia studies. Existing research has mainly focused upon the garden areas that surround care facilities. A smaller body of research has followed people with dementia into public spaces (e.g. Burton and Mitchell, 2006; Sheehan *et al*., 2006; Blackman *et al*., 2007) but much of this work has focused upon functional concerns such as wayfinding and questions of navigability and the legibility of the built environment. In a review of the literature on dementia and neighborhoods, Keady and colleagues (2012) noted a predominance of generic references to “the outdoors” or “urban environment” and were unable to identify any research that had adopted a more multi-faceted understanding of the environment, for example, by taking into account the socio-economic profile of actual places and the implications for residents with dementia. Overall, much of the research on access to the public domain by people living with dementia has been framed by a compensatory-enablement approach to the environment, and in this paper we argue for the benefits of attention to “lived place” (i.e. a more experientially grounded way of understanding the environment) to the debate on dementia and the environment.

## The importance of “lived place”

Out of the critique of a bio-medical model, which has largely ignored the role of the environment, a compensatory-enablement approach to the environment has now become well-established within dementia studies. Based upon mounting evidence that unsuitable and inhospitable environments play a significant role in disabling and constraining people living with dementia, research has focused upon the potential for design of the built environment to alleviate and assuage common symptoms of dementia and thereby to serve a compensatory purpose (e.g. Wilkes *et al*., 2005; WHO, 2006; Van Hoof *et al*., 2010; Marquardt, 2011). Over time, research has broadened to identify environmental features that serve as a resource in achieving the therapeutic objectives of dementia care by promoting independence and enabling people to continue to pursue everyday activities (Day *et al*., 2000; Torrington, 2006; Woodbridge *et al.*, 2016). From this body of research, core design principles have been developed and translated into practice through tools such as environmental audits and design checklists (e.g. Fleming and Purandare, 2010; DSDC, 2011; Waller and Masterson, 2015), primarily intended to promote the legibility and navigability of different settings. This principles-based framework has been adopted internationally in the design of environments intended for the provision of dementia care (e.g. Fedderson and Ludtke, 2014).

Limitations to the compensatory-enablement model include a failure to engage with social and cultural dimensions to the environments inhabited by people with dementia (Day and Cohen, 2000; Marshall and Gilliard, 2014). Commentators have argued that a narrow focus upon physical properties reinforces an artificial division between the social and material aspects of the environment (Keady *et al*., 2012). A further limitation has been the exclusion of people with dementia from the design process and ultimately from the social production of space (Lefebvre, 1991). Heylighen *et al*. (2013) have challenged the principles-based approach to disability within architecture and design on this basis, arguing that it reduces “the human body to a source of an abstracted system of proportions” (p.17). Instead, they argue, architects should acknowledge the “full sensory role” of the body in experiencing the built environment. Through direct day-to-day lived experience a disabled person develops unique insights and knowledge, and this includes the potential to expose an ableist bias within the built environment.

Such concerns within dementia studies have led to growing interest in a “person-in-environment” approach, driven by a more fluid and dynamic understanding of the person–place relationship (e.g. Blackman, 2006; McGovern, 2012; Van Steenwinkel *et al*., 2014). For instance, in relation to the experience of outdoor and public spaces for people with dementia, Brorsson and colleagues (2011) draw on the work of the philosopher John Dewey to develop a transactional perspective, arguing that:

The environment is not just a physical place; it also embraces social, cultural and political aspects and includes spatial and temporal dimensions. Those dimensions are internalized by people who interact with the environment and in turn modify it, creating inseparable connections between them’ (p.588).

From a public health perspective on dementia, Blackman (2006) similarly casts the neighborhood as a multi-faceted people–environment system where spatial, temporal, and experiential factors intertwine. The neighborhood emerges as the result of “walkable zone of experience,” that is given form and structure based upon a person's “walking patterns to nodal points from the home” (p.33). From this perspective, attention to lived place can support a person-centered understanding of the environment.

A focus on lived place has the potential to reveal much about the everyday experience of people with dementia as they venture beyond their front door, helping to understand the capabilities and resources that are vital to remaining socially and physically active within a local community. Hence, a feature of the work by Brorsson and colleagues (2011) has been exploration of how people with dementia manage unpredictable and unforeseen challenges in the public realm. Focusing on examples such as grocery shopping (Brorsson *et al.*, 2013) and pedestrian crossings (Brorsson *et al.*, 2016) their research has underlined the *in-situ* coping, innovation, and resourcefulness of people with dementia in public spaces, but has also underlined the impact of ableist design upon their lives. It is likely then that a focus upon lived place could yield valuable insights relevant to our understanding of social health and its driving ambition to focus upon the capabilities of the person with dementia.

We turn now to introduce the N:OPOP project, showing how our interest in lived place has guided the design of the research and informed our understanding of the environment that frames the study.

## The project

The “N:OPOP” project builds on a pilot study involving 14 carers of people with dementia, conducted in the north-west of England during 2011 (Ward *et al*., 2012). The pilot provided an opportunity to test the feasibility of our chosen methods and to refine the project design in collaboration with stakeholders using workshops that included people with dementia, carers, practitioners, service commissioners, and policy-makers. Through this process we arrived at a qualitative, longitudinal, and comparative design for the present study, framed by a participatory ethos. The study is interdisciplinary, drawing upon critical and environmental gerontology and sociological perspectives on place and space, and is rooted in a constructivist paradigm. As such, we have focused upon the meanings that neighborhood holds for participants as well as their day-to-day neighborhood-related practices.

### Aims and objectives

The main research question is: How can neighborhoods support people with dementia and their carers to remain socially and physically active? And, over the course of five years we have three main objectives:

1. To understand the ways in which neighborhoods support the well-being and everyday lives of people with dementia and their carers.
2. To use our research findings to co-design a locally-tailored and responsive approach to support and community engagement for people living with dementia, their carers and supporters.
3. To implement, pilot and evaluate this model or approach, involving practitioners in facilitating community action in supporting people with dementia to remain active members of their local communities

### Design, recruitment, and methods

There are three fieldsites for the research: The central belt of Scotland (especially the Forth Valley region), Greater Manchester in North-west England, and the county of Ostergotland in Sweden. The different fieldsites will help capture the social and spatial diversity of life with dementia and serve as comparators. In each area, we are recruiting up to 15 co-habiting dyads (not necessarily couples), where one person has a diagnosis of dementia; five people with dementia who live alone and five carers whose partner or relative has moved to a care home. We are asking each couple or individual to participate in three separate interviews:

1. A walking interview, where we ask participants to take us for a walk to or through a place of their choice.
2. A social network mapping interview, where we ask participants to “map” the people in their lives who are important and to describe the nature of each relationship.
3. And finally, we're asking people to take us on a tour of their home, which we are either filming or audio-recording

After an interlude of six–twelve months, we are now in the process of undertaking the network mapping and walking interviews for a second time. Alongside the interviewing, each fieldsite has also recruited an action learning set of eight–ten local practitioners from different sectors and services and a small group of people with dementia and carers, to whom we are feeding back our findings and who will participate in the co-design of the intervention for the second phase of the project.

### Analysis and findings

At present, we have completed the first round of interviews and are now revisiting participants to repeat the walking interviews and social network mapping in support of the longitudinal dimension to the study. Our presentation and discussion of findings here draws on preliminary analysis of interviews with the first 10 to 11 individuals or dyads (each set of three interviews forms a single case study) from the Manchester and Scottish fieldsites. See Table 1 for profile details of the participants included in the preliminary analysis.

**Table 1.** Profile of research participants included in preliminary analysis

	status	age	ethnicity	diagnosis	setting
**Stirling**					
S1	Co-habiting couple	60–65	White British/White American	Alzheimer's	Semi-rural
S2	Carer with partner in care home	70’s	White British	Not known (N/K)	Semi-rural
S3	Co-habiting couple	70–75	White British	N/K	Small town
S4	Co-habiting couple	60–65	White British	Alzheimer's	Urban
S5	PWD and non-resident carer	75–80	White British	Alzheimer's	Urban
S6	Co-habiting couple	65–70	White British	Vascular	Suburban
S7	Co-habiting couple	N/K	White British	Alzheimer's	Village
S8	Co-habiting couple	75–80	White British	Alzheimer's	Village
S9	Carer with parent in care home	40–45	White British	N/K	Urban
S10	Carer with partner in care home	N/K	White British	Vascular	Small town
S11	PWD single householder	65–70	White British	PCA/Alzheimer's	Urban
**Manchester**					
M1	Co-habiting couple	75–80/ 80–85	White British	Alzheimer's	Urban
M2	Co-habiting couple	60–65	White British	Alzheimer's	Urban
M3	PWD single householder	70–75	African Caribbean	Vascular	Urban
M4	Co-habiting couple	75–80	African / White British	Alzheimer's	Urban
M5	Co-habiting couple	70–75	White British	Alzheimer's	Suburban
M6	Co-habiting couple	60–65	White British	N/K	Small town
M7	Co-habiting couple	55–60	White British	Young Onset (Alzheimer's)	Suburban
M8	Co-habiting couple	63–65 / 65–70	White British	Alzheimer's	Urban
M9	Co-habiting couple	70–75	White British	Alzheimer's	Suburban
M10	Co-habiting couple	60–65 / 65–70	White British	Alzheimer's	Urban

Analysis involved open coding and categorizing of over 30 interview transcripts (and filmed footage) from each fieldsite. The interviews, which were transcribed verbatim, lasted between 20 and 120 minutes, and for this paper we have integrated the coding from the home tours, walking interviews, and social network mapping. In the following section, we offer an overview of the emerging themes before focusing in more detail upon examples of the different ways in which people seek to balance their capabilities in a context of available social and environmental resources.

Coded data were organized according to five headings: Person; Place; Connections (i.e. interactions and relationships); Citizenship; and Time. The clustering of data and narratives from the individual case studies serves as thematic headings but also provide an heuristic taxonomy of the different dimensions to the “lived neighborhood” according to the experience of the participants. We explore below how these themes intersect in the lives of people with dementia, creating a dynamic and fluid experience of the local environment.

A second stage to the analysis involved looking across the individual case studies and fieldsites to identify three over-arching or “meta” headings.

1. Diversity/heterogeneity: The research revealed much variance in the situation and context of the study participants, not least in the relationship to their neighborhood. Analysis provided a basis to identify potentially “significant differences” i.e. areas of inequality, or with the potential for inequalities to develop, such as living alone/co-habitation; ageing in place/newly relocated; and differing levels of mobility.
2. Inclusion/exclusion: The meta-analysis allowed us to identify patterns of inclusion and exclusion that might indicate shared aspects of the experience of neighborhood for people with dementia. It also helped us to understand how dementia intersects to create particular experiences of place (e.g. a physically impaired older woman with dementia, who feels unsafe venturing outside alone at certain times of day).
3. Opportunities and barriers: We also identified different types of capability and related strategies essential to coping in the public realm, including examples of how people with dementia exploit and juggle available social and material resources, as well as instances where such efforts were thwarted or undermined within inhospitable environments.

In this next section, we illustrate the interplay of the different dimensions of the lived neighborhood.

### The dialogue between home and neighborhood

Our research shows that the relationship of home to neighborhood is a porous one; as a person's experience of neighborhood alters over time the home can take on new meanings. As we illustrate below, the neighborhood can begin within the homespace, revealing that the two zones are closely intertwined. Hence, for many participants the neighborhood and home were in dialogue, the home was found to enshrine and facilitate people's response to their neighborhood and the demands it made upon them, and so it made sense for our research into neighborhoods to begin where people were “at home.”

For some, the home was a locus for maintaining control and this was illustrated in our interview with Ruth, who described the increasingly exhausting demands of the public realm. Ruth, who lived alone had reconfigured her day and now retired to bed often as early as 5 pm in the evening, describing this as her opportunity to “savour the day.” Long periods spent in bed were a means to counter-balance the effects of keeping up with the pace of life beyond her front-door. For Ruth, the home provided an opportunity to reclaim control by instituting her own temporal frame upon the day. Albeit that she was out of step with the temporalities of a wider world, this enabled her to build and restore the necessary energy reserves to cope with public life:

Interviewer: Are there certain times of the day when you won't go out?

Ruth: I don't go out in an evening. My life. . . my world. . . everybody says it's 24 hours isn't it, but mine is. . . like today it started you know like say with us at half past nine, although I've been up for a few hours. Now by four o'clock normally I would be making to my bed. By five o'clock I'll be lying in bed

I: Is that your usual bedtime?

R: Yeah. I go off balance, because it takes a lot for me to stay upright and to keep myself upright, so. . . I'm safer in bed. Then that's when I do a lot of my planning, my talks. The Ipad, I take photographs and reflect back on what my day was like. And almost savouring the day, and relive the highlights, so it's better than telly

I: But you're still continuing your day, you're just doing it from bed

R: I'm just doing it lying down. And my bedroom you've seen has a big window so you're getting natural light. So you don't feel as if you're in a wee boxed room. But I just know that I'm safe, my door's locked and all's well with the world

Such efforts to control or re-configure time were a significant example of the resourceful and agentic way in which a person with dementia can engage with their environment and were a basis on which to manage life with a degree of independence.

Being connected to a wider world via a view from a window or balcony could also enable a sense of connection to the neighborhood. For instance, June who lived alone in an apartment near the city center talked of the time she spent watching events on the street below her window:

“I spend many an hour sat in my chair watching a fella that works there and honest to god if I ever needed a man to work for me it would be him. He never stops, it's the best worker I've ever seen, he has nobody watching him but what he hasn't done. . . he's painted that building a couple of times and he's redone the floor, I've never seen anybody work like him. So it fascinates me watching him through window.”

Lottie also commented on how she enjoys watching boats on a canal visible from her flat:

L: When the big ships used to come in – and it still happens occasionally – this bridge here will swing round, so all the traffic going to the [shopping] Centre or coming out of the [shopping] Centre has to wait. And we still get occasional big ships coming down here. There was a Norwegian one the other day, flying the Norwegian flag; it goes straight down into Manchester.

I: And you see this from your flat?

L: You can see it from where we are, yes.

Such visual links to the outside world are an example of the dialogue between neighborhood and home that had particular significance for those whose worlds had begun to constrict. However, Nord (2013) argues against reading these more localized lifestyles as necessarily indicators of social withdrawal or decline. Instead, she contends that residents are still living “an active life in their small but quality space” . . . “where they negotiated their own needs, as well as [those of] visitors, and they tried to solve everyday problems with new things and technology” (p.140).What is important here, is that like Nord's participants, the people we visited still possessed a degree of autonomy and self-determination and were able to claim a connection to the outside world based upon their efforts to bring the neighborhood into their home.

Through close attention to lived place, we found that people with dementia uphold a claim upon their neighborhood, managing their environment according to their capabilities and maintaining a sense of self as central to this process. Hence, from a social health perspective practices such as temporalization (i.e. control over time), and strategic positioning toward the neighborhood from within the home all point to active and reflexive engagement with the homespace for people living with dementia as a route to maintaining links with a wider world on their own terms.

### Neighboring and localized relations

Social network mapping provided opportunities to explore the emplaced nature of social relationships. This included insights into types of relationship that have been largely overlooked within dementia studies. Our research shows that neighboring relations were often characterized by reciprocity in a context of managing the tensions between social and physical proximity. The “non-obligatory willingness to take social and practical responsibilities for others” (Abrams, 2006; p25) within the neighborhood was important to many of the participants. For example, Maureen, who lives with her husband in Greater Manchester talked of her appreciation that her neighbors engaged in practical help, putting her rubbish bins (trash cans) out for collection:

M: Yes, they're good neighbours.

I: What makes you say they're good neighbours?

M: Because, the thing is, they bring my bin out for me.

I: Yeah, okay. Up the steep side.

M: Yeah, yeah.

I: And do they do that without being asked, or do you have to ask them?

M: No, no, they do it voluntary. They just do it themselves voluntary. . . [and] they mind our house when we go away

Another couple, Mike and Nora, highlighted a communal concern for vigilance and security when offering a similar definition of the role of a “good neighbor” as individuals prepared to “do a good deed:”

M. I've got really good neighbours.

N: We have got good neighbours, very, very good neighbours.

I: Yeah? That's great.

N: I mean, just as you arrived and I took them dead flowers to the bin, the lad next door, he just stopped me and said, ‘we're going away on Friday’, he said, ‘use the drive if you want’, because what happens, the car in the garage and the van in front of the garage, but also it means that if people in the evening are moving about and they see a car on the drive, they think somebody's in, so all the neighbours, that side and that side and opposite, if they're going away, they let us know, one, for security so we know, and we do the same, and they say to us, ‘use the drive’. It means it's not being empty, so that's what we do.

For others, neighbors are those able to keep a watchful eye, and step in at times of potential risk. Lottie, for instance, spoke of other tenants in her apartment building, including one who stepped in at a point when the condition of her dementia had become a point of concern:

L: The other tenants in here all look after and keep an eye out for each other. Now, it was another tenant that actually shouted for me. . . Yeah, Roger.

I: . . . who actually stopped you from going out?

L: Roger. Because he knew there was something wrong, so he just started shouting for me, sort of thing, which obviously I then went out. But they all look out for each other, so it's more of a safety net.

A number of those we spoke to reported looking out for others as much as they were looked out for themselves. As June explains:

J: He's one of my neighbours and he's a very devout Catholic and he fetches us all a little bottle of holy water with the picture of our Lady of Lordes on the bottle and everybody looks out for him and he's. . . he looks out for everybody else.

I: Do you look out for him, June?

J: Yes, if I thought anybody was going to take the mickey out of him I wouldn't let them. We had one man in there that used to take. . . really take the mickey out of him and when I found out. . .

[later]

I: Is Lily (neighbour) someone else you look out for?

J: Yes, she's. . . when I think about it I look after [lots of people] like that, I think I'm one of the best on me legs and they always knock on my door I don't know why, it's just dawned on me they do and I. . .

I: The neighbours who knock on your door?

J: Yeah. And I am unofficially Lily's carer

What we see in these examples is that while neighboring is important for “emergency” situations, it also forms part of the daily, often mundane activities of everyday life in ways that enable people to continue to maintain a degree of independence. However, people living with dementia are not solely recipients of neighborly support, but rather engage in recursive acts – looking out for other neighbors in ways that have been largely overlooked in research, which has rather narrowly focused upon carer-cared for relationships. Being able to look out for others, and play an active role in ensuring their well-being enables people with dementia to play fuller roles in neighborhood life and in wider communities and constitutes an example of the “everyday citizenship” that was a central theme from our interviews. This capacity to offer care and to watch out for others might be considered a key element to social health, based upon people's ability to participate in social life through reciprocity.

Our analysis echoes many aspects of neighboring research, of non-obligatory willingness, of practical and emotional support, and a complex process of reciprocity within local networks (Bulmer, 1986; Abrams, 2006). We found that neighboring relationships are often characterized by low level, informal, and “benign acts of kindness” (Anderson *et al*., 2014), and companionship (Wellman and Wortley, 1990; Scharf *et al*., 2005) and served as an informal “early warning system” (Wenger, 1990). The reciprocity we observed was at times unequal or “unbalanced” (Thomese *et al*., 2003; van Dijk *et al*., 2013), but does appear to depend on some form of existing familiarity, or (in June's case) being prepared to take a first step. Neighboring is not uniform, nor part of a “communal” structure, but rather takes a more individualized form in terms of the quality of relations that were reported, often constructed through discretion and personal choice.

Alongside neighboring as a form of practical and emotional support, our data reveal a diverse cast of “connections,” people who, while not really “intimates in waiting,” nonetheless help the person living with dementia to maintain a sense of belonging within their neighborhood. These include store keepers and service providers (such as chemists, café and bar staff, and hairdressers), as well as individuals such as dog-walkers or joggers, who may be seen or passed-by on a regular basis. Maureen for example, visits the same café each week with her daughter at the end of a shopping trip:

M: Well, the thing is, is that I always go to [particular shop] and then I go to the supermarket. Then I have a cup of coffee, and it's [daughter] that's with me for coffee.

I: So does [daughter] meet you for coffee or does she take. . .?

M: Yes, she meets me for coffee, yeah. . . . [later] It's a café, yes, yeah. Well, I usually go there after. . . with [daughter], and I also go there with [husband], and when he goes. . . when we go to the Alzheimer's clinic, we go to have lunch there. And I think it's £4.99 for breakfast. . .

I: And is that your favourite place, [coffee shop]?

M: Yeah, that's where I did the 80s there, I was 80. . .

I: Your 80^th^ birthday party?

M: Yes, yeah.

And Viv discussed the connection she had established through regular visits to a local butcher:

V: ‘Our butcher he makes all his own sausages on a Wednesday and it's nice to catch up with, you know, ‘how's the grandchildren?’’

I: ‘Yeah. So he's someone who you have a chat with?’

V: ‘Oh yeah, and I still pop in there even if I don't need anything’

Regular interaction with the same people in the same places builds familiarity and a broader sense of belonging. For individuals living with dementia, our research suggests that this can also build confidence in navigating space. June for example, takes a regular fortnightly trip to her sister-in-law's house around five miles away. She takes two buses, boarding and alighting from the same bus-stop, and uses the trip as an opportunity to also visit a supermarket:

“. . . the bus stop there will take me to [town] and the bus stop on the other side I can go all the way to [shopping] Centre if I want. My sister-in-law lives in between [here and there] and because I've been doing it from my own home I used to catch the same bus I can do that blindfolded. I don't go anywhere else on the bus but I can go to her house. I get the [bus number] there and it stops at [neighbourhood] and then I get a [bus number] and it stops right outside her front door. . . . I can do that no problem. . . I won't get on another bus on me own and I know it's still the same bus going somewhere else but the only place I go on my own is to me sister-in-law's because I've done it for so long it's like going to the [street] corner you know”.

Routine practices have the potential to offer a greater sense of connection to place and establish latent opportunities for support in times of need. Viv recounted a time when, out running alone, she took a wrong turn and became disorientated, and received assistance from staff in a local café:

V: I'm not afraid. Because I know the area quite well, and the people know me and I know them.

I: So you don't get lost when you come out?

V: I have been lost a few times, but that was my own fault because I've took the wrong turning, gone the wrong way, and then straight away, [someone asked me] ‘are you alright love?’ And I said ‘I don't know where I am’, and she was absolutely lovely. She said, come on in and have a cup of tea and calm down.

Regular and routine interaction with “strangers” can enable a level of connectedness beyond people to call on times of crisis. It is important to also recognize the value of mutual recognition and acknowledgment within these informal interactions (Blokland, 2003; Harris, 2008), simple gestures of recognition at a neighborhood level are significant both individually and cumulatively. Such gestures can be as simple as a smile through a window or an acknowledgment of presence when passing-by but can enable a sense of being connected and consequently of belonging in place (Phillipson, 2007).

Clearly then, the neighborhood is not just the physical characteristics of the spaces in which people live, but also how they feel about, identify with and act in their place of residence. Given there is some awareness of the potential contribution social networks make to mental health and well-being in older age (Tomaka *et al*., 2006; Wilson *et al*., 2007), we contend that relations with neighbors and other “proximate strangers” in the neighborhood can play an important role in the social health of people with dementia.

### Place-making and meaning-making about place

In this final section, we consider insights from the walking interviews; *in-situ*, mobile encounters that involved accompanying people as they made their way through their local environment. The method allowed us to observe forms of often unarticulated and embodied knowledge as people moved through the environment as we discussed it, but it also provided valuable insights into the layered and multi-faceted nature of the specific place itself for the people we interviewed.

For instance, Bob took us to a site of Roman ruins at the edge of the small town in Scotland where he had lived since a small child. Here, he was able to point out specific features of the landscape linked to his own biography, such as the dips between hills where he played “soldiers” and fought imaginary battles as a child. The site was an opportunity for Bob to display his in-depth knowledge of local history as he painted a vivid picture of the Roman settlement and of what the excavations had revealed:

‘. . . that's the fort now that we see in front of us. See how it dips down? At the bottom is a burn which we call Rowntree Burn. I think it's still. . I think that's its right name. So the dip you see down there, that's a natural dip. It would've been there, we know that because of the earthworks that you're starting to see now, which is part of the fort on the right here. . .’

These more specific recollections opened out during the walk into a commentary on Scottish national identity and how Bob's pride in being Scottish had informed his politics and worldview. As with many of the participants we walked with, we observed how the intertwined narratives of place and self were prompted by Bob's actual presence in this meaningful setting. Moving through the environment gave structure to his narrative, adding form and substance, which supported Bob to recount stories and anecdotes that he recognized would have been inaccessible to him had he not been speaking *in-situ* in this way:

‘Probably the main reason for wanting you to see this is my short-term memory's going. I find that this place and others like it come to mind quicker now. I'm remembering things now from my childhood that I never thought about for years or literally I'd forgotten. I suppose it's because they've been there so long, still there. . . If you take me to a modern place and asked me to talk like this I'd probably be. . . what do you want me to talk about this for!’

Another significant feature of our focus upon lived place was the opportunity during the walks to see aspects of people's neighborhood identities in action, including examples of everyday citizenship that focused upon the upkeep and guardianship of local spaces. For instance, Mo and Frankie took us for a canal-side walk near to where Frankie had worked most of his life in a local distillery. Their descriptions of the neighborhood were punctuated with expressions of their fondness and attachment to the area and of their pride in it. Walking along we came across a fire on the bank of the canal and a small group of children nearby. At first the couple express their concern but then swing into action:

M: Why have they got a fire here? I don't like that, it shouldn't be

I: Are the kids burning something?

M: Youngsters, as long as it doesn't. . . when you think what's behind it, there's whisky here, you just don't start fires here. . . Maybe we should have a look Frankie? They shouldn't be burning anything around this area. These warehouses are full of whisky [The couple ask passers-by if they have a water bottle on them to help douse the flames] It's so dry here it could just take alight

I: [To child] You could get your trousers caught son, I would come out of there

M: Mind yourself. . . I'll tell the distillery. We'll go back and get somebody to come and sort it. Aye, it's burning itself out hopefully

Moments like this were valuable in offering glimpses into how people tackled the unpredictable and unforeseen events that often punctuate our experience of local areas. We saw how Mo and Frankie worked together supporting one another, expressing a proprietorial and protective concern for their neighborhood and acting to watch over the children they had encountered. In this way, different capabilities were enacted for us to witness in the context of this unplanned situation, offering insights, which would have been inaccessible to us had we relied upon more traditional sedentary interviewing.

Close attention to lived place was also an opportunity to better understand the type and nature of the barriers that people faced at a local level. Ruth (mentioned earlier) lived on a main road in a suburb of a large Scottish city. She described how the road had become increasingly busy with traffic over the years and now represented a major obstacle. Walking along the road the interviewer tried to put into words how it felt to follow the narrow pavement as large articulated lorries sped past:

Interviewer: So these. . . when these lorries come, it's quite a major thing isn't it, they almost sweep you up in their backdraft. . and sweep you along. [Lorry passes] There's loads of them isn't there. Huge big. . . and it's loud and it kind of pulls you along a bit

In response Ruth described her own embodied experience of the road:

Ruth: Yes. . . and when you were saying about the backdraft I nearly got knocked down but it was. . . I was almost drawn into the road and I couldn't do anything about it. And it was because I wasn't concentrating. When I'm walking I need to concentrate. You take it for granted, you walk and you know you can, but I've got to make sure and I'm constantly aware. Feet on the ground, splay your feet out, you've got to be well grounded. Walk towards the thingmy [fence] and if you weren't with me I'd be holding on

Reminded of an occasion when she was almost caused to stumble into the line of traffic Ruth makes a point about the distinctive experience of urban walking for herself as a person with dementia as compared to the researcher who accompanies her: “you take it for granted, you walk, and you know you can.” As she makes clear, there is much about the day-to-day experience of outdoor and public spaces that are “taken for granted,” naturalized and hence considered unproblematic. Inhabiting this traffic-heavy space requires a particular way of being: “constantly aware,” “splay your feet,” “got to be well grounded,” that involves how she holds herself, her posture, gait and mental focus as she concentrates to avoid being pulled over. Her bodily experience is central to her relationship with the environment and this setting makes clear demands upon her; to walk the busy road is an effortful and ultimately tiring endeavor.

Finally, we turn to the theme of “place-making” as a key example of how people with dementia balance their own abilities and capabilities with environmental resources. Our interviewing revealed many instances of reflective and knowing efforts being made to shape neighborhood spaces into supportive and meaningful places. Place-making offers a way of capitalizing on a local community's assets and potential, with the intention of creating public spaces that promote people's well-being through active participation in place (Pierce *et al*., 2011). Such place-making was at the heart of Rita's attempts to show us how she has striven to adopt her neighborhood as a social space better equipped to support her needs:

R: There're several shops that I've really got to know the owners and I think that's part of dementia awareness. It's a personal approach. It gets you far more commitment from businesses and people. You know, I just pop in, like. . . well there're too many to name really but one is a café that is very dementia friendly. I mean I'm working on the toilets, and that, but we'll get there, but they are just so dementia friendly, you know, and the tables really spread apart and it's an open, light area, carpet that doesn't do your head in. It's just everything that. . . you know, I wouldn't hesitate in taking my friends with dementia in there.

I: And is that somewhere that you go socially, that you'll go and have a break for a coffee?

R: I do, yeah, because it's central. It's quite close to the hair salon and it's quite close to some of the other shops that I pop in. I tend to try to pop in, even if it's just to say hi, just to keep that contact because I feel it's no good just going in and saying, you know, can you read the leaflet about becoming a dementia friendly shop, and then just leaving them to it. I've got to. . . I must admit I've been known now in the town.

Central to many such examples of place-making was how people used the disclosure of their own diagnosis to raise awareness in settings that they frequented. For example, Ruth described the impact of disclosing her diagnosis upon her local library service after many months of problems:

R: So my library was a bit of a. . I knew there was a problem but I just didn't know what it was and it took ages before it got to a crescendo where suddenly there's a problem. And then when I. . . my. . . I just took it upon myself and went up to the girl and said ‘look I'm tired of getting these letters. And they're threatening in nature. I've been a member of this library since I was a wee lassie. Why would I now be getting fined?’ You know. And then I said you know, ‘I've got a diagnosis of dementia’. Just tears were streaming and I'm crying because. . . and it wasn't crying, you know just tears, emotional. I says ‘is there not something you could put on the system’ you know ‘cause I want to use the library, I've always used the library’. Why would I stop using the library!

I: So from the library's point of view, they've. . .

R: They've come up trumps. Now they're very aware. . . they notice with others and they're paying a wee bit more attention. So it's raising awareness. And that's what I feel I've done all the way through my dementia

Another, perhaps less dramatic, but no less important process of place-making happens more subtly and cumulatively as individuals navigate through, and become increasingly familiar with a particular place. Viv, for example, talked of the importance of a woodland walk for her and her grand-children:

V: There's a tree that its name is the ‘knock knock’ tree because somebody has painted a white door and has put a tiny little door knocker on it. So you knock on the door to see if the fairies are in, and of course they never are, they've always gone out to see to fairy business.

I: So do they want. . . every time they [grand-children] come that's something they want to do?

V: Yeah.

I: So are they ever hopeful that the fairies will be there?

V: Yeah, the ‘knock knock’ tree.

I: That's really special; building special memories with them up here, aren't you?

V: Yes, and the seven year old granddaughter loves going on adventures and so as soon as we're in the valley she'll say, where are we today, grannie, and I have to think. So I've turned it round and say, well where do you think we are, and we have all sorts of adventures. We're in such and such a different land and what have we got to look out for and are they goodies or baddies, and I put on different accents and voices

Our analysis indicates that there are many approaches that people living with dementia employ to shape place. Memories and imagination as well as routine practices, and being connected to others are all ways in which neighborhoods are not just experienced, but also come to be constructed, and which may be supportive for social health. Ultimately, these narratives of how people's actions impact upon place, or which serve to convert non-descript space into knowable place, signal the dynamic, and transactive relationship that people have with their neighborhood. Within a context of understanding social health, place-making emerges as a significant capability in creating the conditions not only for managing life with a degree of independence but also in fulfilling potential and local obligations through such everyday forms of citizenship.

## Concluding remarks: the neighborhood as a context for social health

In this paper, we have used a “lived place” lens to explore the environmental challenges that people with dementia face at a local level. We have shown that the neighborhood provides a significant arena for people to draw upon their personal potential and capabilities in order to compensate for the limitations they experience. In this respect, the study supports a key argument made by Vernooij-Dassen and Jeon (2016) that “Social health involves making a dynamic balance between opportunities and limitations, affected by external conditions such as social and environmental challenges” (p.701). The findings thereby suggest that neighborhoods have a vital role to play in preventing or reducing disengagement and are a primary locus either to enable or constrain the key features of social health for people living with dementia.

Our research demonstrates that a focus on lived place offers a way to get to know people both “in place” and “through place.” The participants defined themselves according to the places they lived and spent time; they used the material environment as a means to articulate aspects of identity and selfhood; and offered insights into their values, beliefs, and sense of belonging through the diverse spaces they chose to inhabit. As such, the study provides a compelling argument for greater environmental awareness within dementia care, not simply as a compensatory mechanism to address the symptoms of the condition but as a hitherto largely untapped resource in person-centered practice. This leads us to the recommendation that getting to know the person-in-place is an important consideration for practitioners in seeking to foster social health.

Spending time with people in the environments where they live, watching as they move about and interact, gave us direct insights into the broad spectrum of socio-spatial capabilities and strategies that people employ in the course of day-to-day living. Indeed, a focus on lived place appears particularly well suited to foregrounding a capabilities-led approach to dementia, one which lies at the heart of social health as outlined recently by Vernooij-Dassen and Jeon (2016). Our research has begun to capture capabilities and capacities in action, showing how people work in collaborative and innovative ways and call upon intra- and inter-personal resources “in-the-moment” as the neighborhood makes demands in ways that can be unpredictable and unforeseen. The findings point to the scope of what people with dementia are able and enabled to do in their neighborhood rather than merely what is no longer possible. Hence, dementia care practice may benefit from mobile methods of assessment and interviewing as a route to deepening our understanding of the person as a foundation to person-centered practice.

However, there is immense diversity within participants’ relationship to their environments and our findings suggest that certain types of difference may be significant in that they could lead to relative disadvantage or inequalities between people with dementia. For example, living alone and venturing into the public realm unsupported can deplete energy reserves and ultimately prove exhausting. While, a long-standing relationship to a particular locale can bring benefits not only to independent movement within it, but also in shoring-up a sense of self and identity. In a context of growing recognition of the health inequalities that exist between people with dementia (Cooper *et al*., 2016), our research thereby adds an environmental dimension by underlining how differing relationships with the neighborhood can lead to diverse outcomes and potentially to inequalities in social health. Identifying and responding to these areas of “significant difference” could help to shape professional practice and neighborhood-based interventions in support of social health. Hence, policy on dementia needs to disaggregate the category of “people with dementia” not least in our understanding of what makes a particular environment “dementia-friendly.”

We have argued for the importance of attending to types of relationship and forms of support that remain overlooked according to an existing narrow focus upon carer/caree dyads within dementia studies. Even fleeting ephemeral encounters have a role to play in supporting the social engagement of people with dementia, fostering a sense of belonging, and social connectedness. Our research highlights the importance of understanding how emplaced social networks can work as a system of support, and we have outlined forms of socio-spatial attachment and involvement, which ultimately help to limit the risk of disengagement and thereby to support social health. We suggest there is a need for greater attention to these more diffuse forms of support within dementia studies alongside a better understanding of how the wider networks, in which people with dementia are positioned, work to support them.

Finally, the study has begun to reveal how the neighborhood can help people to fulfill their potential and perceived obligations at a local level. The research to date has uncovered many different examples of everyday citizenship, those acts, and practices, whereby people make contributions to the places they live and the networks to which they belong and display an awareness of their rights and responsibilities in doing this. Building on the debate over social citizenship, Bartlett (2016) has similarly underscored the importance of recognizing “citizenship within the practice of the ordinary” in a context of living with dementia. A particular example highlighted here is place-making, through which people with dementia appropriate local spaces often seeking to render them more dementia-inclusive. Across research, policy and practice, we can learn a great deal from these acts of place-making, partly because they illustrate the preferred or desired environments that people with dementia wish to inhabit, but also because they demonstrate the attributes and qualities of an environment that are vital in order for social health to flourish.

## Ethics

This research received approval from the Social Care Research Ethics Committee: **15/IEC08/0007**

## Conflict of interest

None.

## Description of author's roles

Richard Ward: Work Program Lead, Formulated research questions, designed study, project management, analyzing data, lead author of paper. Andrew Clark: Work Program Deputy, Formulated research questions, co-designed study, Manchester lead, analyzing data, co-author of the paper. Sarah Campbell: Development of research tools, conducting fieldwork (Manchester), analyzing data. Barbara Graham: Development of research tools, conducting fieldwork (Stirling). Agneta Kullberg: Development of research tools, conducting fieldwork (Linkoping). Kainde Manji: Conducting fieldwork (Stirling), analyzing data. Kirstein Rummery: Mentor to Project lead. John Keady: Mentor to Project Deputy, Lead of Research Program.
